# From the laboratory to the field: how to mitigate pregnancy losses in embryo transfer programs?

**DOI:** 10.1590/1984-3143-AR2024-0032

**Published:** 2024-08-12

**Authors:** Marcelo Marcondes Seneda, Camila Bortoliero Costa, Amanda Fonseca Zangirolamo, Mariana Moreira dos Anjos, Gabriela Rodrigues de Paula, Fábio Morotti

**Affiliations:** 1 Universidade Estadual de Londrina, Laboratório de Reprodução Animal, Londrina, PR, Brasil; 2 Instituto Nacional de Ciência e Tecnologia do Leite – INCT Leite, Londrina, PR, Brasil

**Keywords:** embryo mortality, embryo classification, recipient selection, evaluation of corpus luteum, paternal effect

## Abstract

Pregnancy losses negatively affect the cattle industry, impacting economic indices and consequently the entire production chain. Early embryonic failure has been an important challenge in the embryo industry because proper identification of embryo death at the beginning of gestation is difficult. This review aimed to provide a better understanding on reproductive failure and the relationship between early embryonic loss and different reproductive biotechniques. This review also considers insights and possible strategies for reducing early embryonic loss. The strategies addressed are as follows: i) great impact of rigorous embryo evaluation on reducing embryo losses; ii) selection of recipients at the time of transfer, taking into account health and nutritional status, and classification of the corpus luteum using ultrasound, either in area or vascularization; and iii) paternal effect as one of the factors that contribute to pregnancy losses, with a focus on embryo transfer.

## Introduction

Cattle farming is of great economic importance both globally and nationally. Despite the importance of cattle farming, reproductive failure remains a substantially managerial and economic challenge. Globally, reproductive inefficiency are estimated to cost the cattle industry more than US $ 1 billion annually (USDA, 2009, 2010). Although several factors influence reproductive efficiency, the main cause is gestational loss (Perkel et al., 2015), which reinforces the need for a greater understanding of the subject.

The period of gestational loss in cattle is generally classified as early embryonic mortality (EEM), when it occurs between fertilization and 28 to 32 days after fertilization, late embryonic mortality (LEM) when it occurs between days 28 and 30 and up to 45 days, early fetal mortality when it occurs between days 45 and 60, and late fetal mortality from day 60 until the end of gestation (Smith et al., 2022; Albaaj et al., 2023). In cattle, gestational losses are more prevalent in the embryonic than in the fetal stages (Santos et al., 2004), have variable causes (Diskin and Morris, 2008; Farin, Piedrahita, Farin, 2006; Cheng et al., 2016; Pohler et al., 2016; Abdalla et al., 2017) and are often undetermined (Santos et al., 2004).

A summary of the incidence of gestational losses has been reported in specific meta-analyses of dairy cattle (Albaaj et al., 2023) and beef cattle (Reese et al., 2020); however, several factors, including the animal's production category, stage of pregnancy, and environmental conditions, influence gestational losses (Diskin et al., 2016; Fernandez-Novo et al., 2020).

In dairy cattle, an average of 27% of gestational loss occurs in the early embryonic period (Albaaj et al., 2023) and can reach 40% in cows with moderate production (Diskin et al., 2016), 12% (ranging from 3.5 to 25%; Wiltbank, et al., 2016; Santos et al., 2004) in the late embryonic period, 7% in the early fetal period, and 2% in the late fetal period (Albaaj et al., 2023). In beef cattle, an average of 28.4% of gestational losses occurred up to day 7 of gestation, 3.9% occurred between days 7 and 16, and 15.6% occurred between days 16 and 32, totaling 47.9% of losses in the early embryonic period. Therefore, 6% of gestational losses occur after the first month of gestation (Reese et al., 2020) and can vary between 5 and 20% (Perry et al., 2005; Wiltbank et al., 2016). Although the periods investigated vary among studies, the results provide a summary of the percentages of cattle losses and highlight their biological and economic challenges, especially concerning embryonic losses. Therefore, it is important to discuss the relationship between embryonic losses in different reproductive biotechniques (Baruselli et al., 2010; Reese et al; Hansen, 2020).

In addition, because the highest percentage of gestational losses in cattle occurs in the embryonic phase, detecting these losses, especially by diagnosing them at 30 and 60 days, offers an opportunity for timely reproduction and reduces the costs associated with this source of reproductive inefficiency (Reese et al., 2020). Currently, there are several techniques for detecting gestational status, such as transrectal ultrasound, circulating concentrations of progesterone, circulating placental products (pregnancy-associated glycoproteins (PAGs) and microRNAs), and interferon-stimulating gene expression in peripheral blood leukocytes (Ealy, Seekford, 2019).

Transrectal B-mode ultrasound is a highly accurate method and widely used for gestational diagnosis. However, its use is limited to after–27th-28th day of gestation (Nation et al., 2003). Pregnancy-associated glycoproteins expressed in the third week of gestation in cattle (Wallace et al., 2015) have also been shown to be accurate for commercial diagnosis, but are limited to the 28th day of gestation (Ribeiro et al., 2014; Pohler et al., 2016), and their concentrations are associated with the likelihood of maintaining pregnancy (Pohler et al., 2016). Therefore, Doppler ultrasound has been proposed as an accurate method for identifying nonpregnant females from the 20th day of pregnancy (Siqueira et al., 2013; Pugliesi et al., 2014; Melo et al., 2020).

Given the importance of reproductive loss, it is necessary to develop strategies to reduce pregnancy failure. This review aimed to provide insights into possible strategies for reducing embryonic loss after embryo transfer.

## Reproductive biotechniques *vs.* pregnancy loss

The use of biotechniques as a reproductive management tool has evolved considerably in recent decades, resulting in increased reproductive efficiency in dairy and beef cattle. Despite the increase in reproductive ability, provided to the herd through reproductive biotechniques such as artificial insemination (AI), fixed-time artificial insemination (FTAI), embryo transfer (ET), and *in vitro* fertilization (IVF), understanding the factors related to their success during application is necessary. In this context, pregnancy rates were obtained, mainly the rates of pregnancy loss.

Several studies have investigated the influence of *in vivo* and *in vitro* methods of embryo production and cryopreservation on pregnancy rates and embryo loss. IVP blastocysts have many characteristics that differ from those of blastocysts produced *in vivo* by superovulation, such as accumulation of intracellular lipids, oxygen consumption, DNA methylation, and gene expression (Burdge, Lillycrop, 2010; Rizos et al., 2002; Reese et al., 2020; Banliat et al., 2022).

The reproductive failure rates in beef cattle described in a meta-analysis were 32.2%, 49.5%, and 54.6% for cows subjected to AI after the natural expression of estrus, FTAI, and ET, respectively (Reese et al., 2020). Reportedly, a reduction of up to 24% in the pregnancy rate for embryos produced *in vitro* compared to *in vivo* (Farin, Crosier, Farin, 2001) and 7.4% lower pregnancy rate for recipients who received cryopreserved embryos (frozen or vitrified) than fresh ones (Hansen, 2020). The status (fresh or cryopreserved) of the embryo also tends to influence gestational loss (17.2% *vs.* 22.3%; Baruselli et al., 2010). ​​This corroborates the findings of Stewart et al. (2011) and Demetrio et al. (2007),who observed greater gestational loss after the initial diagnosis in both *in vitro* and *in vivo* ET than in AI.

In a study conducted by Ealy, Seekford (2019) on embryos produced *in vitro*, pregnancy rates ranged from 29.5% to 54.3% with an average of 40.1%. In contrast, for embryos produced *in vivo*, pregnancy rates were higher, ranging from 41.5% to 90% with an average of 64.1%. Furthermore, the pregnancy loss rates in IVP embryos range from 5% to 7% after 90 days of pregnancy. Surprisingly, only 27.1% of the cows that received IVP embryos were able to maintain pregnancy until term. Moreover, early embryonic loss (32 d) after fixed-time embryo transfer (FTET) in dairy cattle ranges from 53% (Diskin et al., 2006) to 58% (Pereira et al., 2016).

Notably, bovine embryos are normally transferred to recipient females approximately seven days after estrus or early ovulation when the embryo has reached the blastocyst stage of development. Therefore, most biological, physiological, and technical causes of a female's failure to produce a blastocyst seven days after natural insemination or AI are avoided when a blastocyst-stage embryo is transferred to a female (Hansen, 2020). Therefore, ET recipients are expected to have higher pregnancy success rates than inseminated females. However, except for heat stress or in cases where the female is a repeat breeder, in the absence of these infertility factors, the percentage pregnant cows after ET is generally equal to (or only slightly higher than) AI (Carter et al., 2008; Sartori et al., 2002; Wiltbank et al., 2016 - data compilated by Hansen 2020).

In the following topics of this review, we addressed tools or alternatives to improve pregnancy success after ET, allowing the embryo's competence for survival and maternal capacity to support embryonic development. Among them are the production of a better embryo (with rigorous quality and stage classification) and improved uterine receptivity, taking into account the nutritional and health aspects, as well as the characteristics of the corpus luteum. In addition, we also examined the paternal effects on transferred embryos.

## Strategies for minimize embryonic mortality

### Embryo selection: different strategies to reduce losses

Observing the comparative results between embryonic losses derived from transferred embryos and conception rates by AI/TAI, we observed differences. Thus, efforts have been focused on reproductive biology research to determine the morphological, cellular, and molecular characteristics involved in the successful development of pregnancy. These efforts have advanced our understanding of the mechanisms for evaluating embryos transferred to recipients. Furthermore, these pregnancy losses typically occur in the first few weeks after ovulation or embryo transfer and are, on average, 40%–60% (Wiltbank et al., 2016). Environmental factors can interfere with gametes and embryos quality; therefore, events occurring pre and post ovulation can affect the development of gametes, zygotes, and embryos (Denicol, Siqueira, 2023). Compared with embryos produced *in vivo*, pregnancy rates of *in vitro* embryos are 10–40% lower than those of embryos produced *in vivo* (Ealy, Seekford, 2019; Reese et al., 2020).

In *in vitro* production, we evaluated the stages of development; however, oocyte competence occurred when the pre-ovulatory follicle and the oocyte itself needed to complete a series of cellular events (Blondin et al., 2002), such as the accumulation of organelles and maternal mRNA (Krisher, 2004), in addition to the resumption of meiosis (Franciosi et al., 2014). Previous studies have shown that oocytes that mature *in vivo* are more competent in supporting embryonic development than those that mature *in vitro* (Rizos et al., 2002). These differences may be due to differences in gene expression, transcription, and protein abundance in early embryos derived from these oocytes (Banliat et al., 2022).

Oocytes play an important role in initially supplying the embryo with mRNAs and organelles during maternal-to-zygotic transition. Therefore, the oocyte quality and changes in oogenesis and/or maturation can affect embryonic development. Furthermore, the presence of miRNAs is also important. MiRNAs are small non-coding RNAs that regulate gene expression after transcription. miRNAs are present in transcriptionally quiescent mature oocytes and preimplantation embryos and exhibit low levels of transcription prior to embryonic genome activation (Telford, Watson, Schultz, 1990; Misirlioglu et al., 2006).

In a recent study, 935 unique targets were identified in bovine embryos using 73 miRNAs on days three and five of development. Gene ontology for these targets showed enrichment for 54 terms, the most substantial being RNA polymerase II transcription, cell cycle, cell maturation, and most notably, stem cell differentiation (Paulson et al., 2022). These results indicate the essential role of miRNAs in bovine preimplantation and embryonic development.

In this context, pre-implantation embryos are extremely sensitive to the environment, resulting in changes in their development (Wooldridge et al., 2022). Moreover, it is unremarkable that conditions during the preimplantation period can exert short-, medium-, and long-term effects on the embryo. This period of development involves a multitude of events that set the stage for future progression of pregnancy (Denicol, Siqueira, 2023). Epigenetic markers, such as DNA methylation, are lost and reinserted during reprogramming for embryonic implantation under the influence of the environment (Burdge, Lillycrop, 2010). The first cleavage division, degradation of maternal mRNA, minor and major embryonic genomic activation (Hamatani et al., 2004) and, differentiation of extraembryonic tissues (Grazul-Bilska et al., 2011), are among the distinct events that occur during preimplantation and can be affected by environmental signals. Finally, changes in the peri-ovulatory microenvironment, highlighting follicular fluid (Sohel et al., 2013; Ávila et al., 2020) can have impacts during oocyte and embryonic development.

In bovine embryo transfer protocols at the blastocyst stage, embryos are normally transferred to recipient females approximately seven days after estrus or early ovulation. Once this embryo is transferred, all possibilities of the cow failing to produce blastocysts are covered, unlike in AI/TAI. However, the pregnancy rates are generally similar between ET and AI. Factors intrinsic to the recipient female are discussed in the following sections. Furthermore, factors related to embryo production stages and the means used, among other factors, can directly reflect the production rate and maintenance of embryo pregnancy *in vitro*. The success of maintaining a pregnancy after ET depends on the creation/selection of an embryo of extreme quality, improving the uterine receptivity of the recipient female, and optimizing the creation of new tools for the production and transfer of embryos (Hansen 2020).

Embryos produced *in vitro* have a lower blastocyst yield per oocyte and lower embryo quality, contributing to greater sensitivity to cryopreservation, compared to those produced *in vivo*. At the beginning of IVM, the intrinsic quality of *in vitro* maturation is related to up to 60% of the failure to advance to the blastocyst stage. However, events that determine embryonic quality occur between the zygote and blastocyst stages (Lonergan et al., 2001). Despite the advances in *in vitro* biotechniques, processing causes stress to oocytes and embryos, thereby affecting their development (Melo-Sterza and Poehland, 2021). Characteristics such as darker cytoplasm, a greater proportion of lipids from the TAG class (Abd El Razek et al., 2000), vacuoles in trophoblastic cells, changes in intercellular connections (Fair et al., 2001) and greater fragility of the zona pellucida (Duby et al., 1997) have been reported as differences between *in vitro* and *in vivo* embryo production.

Physiologically, free radicals have pronounced effects on DNA, RNA, and protein synthesis; however, they can alter the cell membrane, increase the intracellular pH, and interfere with mitochondrial function (Dias, Pivato, Dode, 2017). Embryos produced *in vitro*, mainly in high-oxygen tension systems (20%), suffer from an imbalance in the production and/or accumulation of ROS, which is characterized by oxidative stress. This imbalance can have harmful effects on embryonic development, including metabolic changes (Pawlak et al., 2024), reduction in ATP levels, lipid peroxidation, changes in protein synthesis, membrane permeability, and mitochondrial and endoplasmic reticulum function (Cagnone and Sirad, 2013; Yoon et al., 2014).

However, from all the changes comparing IVP embryos to *in vivo* embryos, it is well established that better quality embryos result in higher pregnancy rates than lower quality embryos. Therefore, if embryos can be accurately evaluated and high-quality embryos can be selected for ET, the commercial value of the selected embryos would increase, with a subsequent decrease in the number of recipients required and an overall improvement in ET efficiency. Therefore, evaluating the embryo before transferring to the recipients is extremely important especially when this embryo is being used post freezing (Bó, Mapletoft, 2018).

To date, the most common way to determine embryonic quality has been through morphological assessments using stereomicroscopy (Hansen, 2020). However, because embryonic quality is based on visual analysis, it varies between observers, has low reproducibility, and can be influenced by the observers (Rocha et al., 2017). Due to the subjectivity of this assessment, differences have already been observed in the ultrastructure (López-Damián et al., 2008) and the blastocyst transcriptome determined similarly using microscopy has already been observed (Driver et al., 2012). Thus, an ideal scenario would be a large-scale use to discover possible markers of embryonic quality with the aim of predicting post-transfer embryo survival and pregnancy outcomes. Consequently, a wide variety of methodologies, from optical methodologies to methodologies based on omics assessments, have been used to evaluate and analyze bovine embryos (Rabel et al., 2023).

Notably, the best-quality embryo must be selected with quality grade I (1: Excellent or Good. The embryos have a symmetrical and spherical mass with individual blastomeres that are uniform in size, color, and density, see in IETS, 2020) and/or grade II quality for transfer to a recipient, and selection must be applied when this embryo goes through the cryopreservation process. Farin, Slenning, Britt (1999) concluded that embryos selected for better quality had higher pregnancy rates. Additionally, they highlighted the differences and subjectivity between the types of evaluators when selecting embryos (evaluated under a stereomicroscope). The difference in conception rates between the selected embryos with quality grades I and II in *in vitro* embryos of approximately 17% in heifers and 22% in lactating cows has already been observed, with the highest percentage being for quality grade I (Demétrio et al., 2020); while this difference in embryos transferred *in vivo* was 44.15% and 32.58%, for grade I and grade II, respectively, after 30 days of transfer into Simmental cows (Erdem et al., 2020).

Additionally, there have been many questions about the best developmental stage for embryo transfer to the recipient. However, the vast majority of studies have shown that the developmental phase does not have much influence, but when evaluating embryonic quality, it is relevant at these stages. (Coleman et al., 1987; Hasler, 2001; Bényei et al., 2006; Ferraz et al., 2016; Erdem et al., 2020). According, to Putney et al. (1988), the lowest pregnancy rate was achieved after the transfer of embryos at the morula stage; however,, there was an increase in the pregnancy rate according to embryonic development in the blastocyst phase, initial to expanded blastocysts.

Hasler et al. (1987) determined that the highest pregnancy rate was achieved after transferring embryos at the early blastocyst and blastocyst stages and that both stages of development were associated with a higher pregnancy rate than the compact morula and expanded blastocyst. When the quality of the structures was evaluated, similar results were obtained: a higher pregnancy rate in the initial blastocyst and blastocyst. Furthermore, Park et al. (2023) showed that pregnancy rates in Hanwoo (*Bos taurus* coreanae) cows varied according to the stage of embryonic development of embryos transferred *in vivo* (67.86% vs. 63.49% for morula stage; 64.00% vs. 54.72% for early blastocysts; and 50.00% *vs.* 47.83% for blastocysts in fresh *vs.* frozen/thawed embryos, respectively). This difference between the results clarifies to technicians that particularities are still highly expressed in each situation/reality evaluated in the field. More studies and standardization of protocols for embryonic culture according to the different subspecies, categories, and conditions of animals are necessary.

In this context, to enhance the embryonic quality and development analysis, one of the most useful optical options with satisfactory results during bovine embryos evaluation is the Time-Lapse Monitoring (TLM) method; however, this method has not been used on a large-scale bovine production. Since embryonic development is a dynamic process, critical developmental phases may go unnoticed with traditional and unique morphological assessments (Rocha et al., 2017; Angel-Velez et al., 2023; Magata, 2023). In addition, some studies have observed that morphokinetic indicators (MKIs), such as the timing of first cleavage, number of blastomeres at first cleavage, and number of blastomeres, could be used as markers to predict blastocyst quality and pregnancy outcomes (Sugimura, Akai, Imai, 2017). Some researchers have even proposed that MKIs would be a superior solution to replace the IETS morphology-based classification systems (Rabel et al., 2023).

Since biomarkers are detected in-omics technologies, especially related to transcriptomics and metabolomics, presence of markers related to embryonic quality are also suspected (Saadi et al., 2014; Herrera, 2016; Mullaart, Wells, 2018). However, during field applications of these technologies, more questions are raised than provisional solutions. For example, if a transcriptomic or metabolomic assessment is used for evaluating embryos in the field, it would be possible to perform a biopsy and extract RNA from the entire embryo produced in each *in vitro* production cycle. Additionally, analysis of all the results in terms of time for transfer might be possible; however, this process is not feasible.

Another way of evaluating embryonic quality is through evaluating the culture medium. However, for reliable evaluation, embryos should be cultured individually–a method used in basic research for metabolomic evaluation. Therefore, although these tools are powerful and useful for understanding pre-implantation embryo development/physiology, their large-scale, field-based applicability is nearly impossible. Hence, traditional evaluation methods, such as selection based on the morphological aspects of stereomicroscopy, are still used. Furthermore, it is expected that innovations will occur to optimize and make new possibilities accessible for embryo evaluation and enhance conception rates.

### Recipient selection

An inadequate uterine environment can lead to pregnancy loss even before the 7th day in an inseminated cow or after the transfer of a good embryo. Therefore, the selection of recipients is equally relevant to the selection of the donors and embryos to be transferred. In 1998, McMillan estimated that only approximately 40–50% of recipients were able to maintain the pregnancy. Other studies have shown that cows exhibit the same behavior after receiving several embryo transfers, with some resulting in pregnancy and others having difficulty getting pregnant or becoming empty (Geary et al., 2016; Moraes et al., 2018). Further evidence of uterine receptivity problems as a cause of infertility was the finding that 32.4% of recipients, who received five embryos, were unable to become pregnant with any embryos (Martins et al., 2018). Other factors directly interfering with pregnancy after the embryo transfer include low body condition scores (Wallace et al. 2015), adequate health management (Aono et al., 2013), lactation period of dairy cows (Hasler, 2001; Ferraz et al., 2016), temperament, consequent management stress (Kasimanickam et al., 2018, 2019), and metabolic changes and diseases in the pre-and postpartum periods (Ferraz et al., 2016; Ribeiro et al., 2016; Barbosa et al., 2018; Estrada-Cortés et al., 2019).

### Sanity management

The presence of reproductive diseases in cattle herds is the main obstacle to the expansion of global livestock farming. Approximately 50% of embryonic deaths are linked to infectious diseases (Aono et al., 2013). Questions have been raised regarding the potential risk of infection and the pathogenic effects of infectious diseases on preimplantation embryos since the advent of the commercialization of embryo transfer (Fray et al., 2000).

Bovine alpha-herpesvirus 1 (BoHV-1) and bovine viral diarrhea virus (BVDV), the etiological agents of infectious bovine rhinotracheitis (IBR) and bovine viral diarrhea (BVD), respectively, exert a notable influence on pregnancy loss in cattle (Aono et al., 2013). BoHV-1 can directly affect ovarian functionality and embryonic quality, in addition to compromising oocyte viability (Bielanski et al., 2013). Furthermore, infections that occur before conception result in viral invasion of maturing oocytes within the follicles (Rufino et al., 2006). BVDV reaches reproductive tissues, interferes with follicular and embryonic development, and persists for weeks in the ovaries of infected females (Rahim-Tayefeh et al., 2023). Moreover, BVDV can infect embryos through donor females, reaching biological materials related to the technique (Bielanski et al., 2013). Furthermore, infection with this virus is associated with ovarian hypoplasia in persistently infected cattle (Grooms et al., 1998), delayed follicular growth (González Altamiranda et al., 2020), and reduced ovulation rates in response to treatments. superovulation (Kafi et al., 1997).

The interaction between BVDV biotypes and the oocyte zona pellucida affects the rate of early embryonic development during *in vitro* fertilization (Rahim-Tayefeh al., 2023). Briefly, BVDV can modify the expression of genes relevant to fundamental biological processes through the activation of signaling pathways, transcriptional regulation, and interference in the cellular microenvironment during persistent fetal infection (Hansen et al., 2010).

Neosporosis, a disease caused by the parasite *Neospora caninum* that promotes infection in cattle, is one of the main causes of abortion and consequences for the embryo, birth of weak calves, neurological problems, and pregnancy loss in livestock (Rodríguez et al., 2022). According to Pessoa et al. (2016), cows seropositive for *N. caninum* have a higher incidence of pregnancy loss (35–270 days post-artificial insemination) than seronegative cows. Furthermore, *N. caninum* DNA was detected in 44.4% of the aborted fetuses from seropositive cows. In a study by De Souza et al. (2022), cows with a history of abortion were 2.3 times more likely to be seropositive for *N. caninum*. In addition, approximately 47.06% of females with a history of reproductive disorders tested positive for neosporosis, indicating an association between the infection and reproductive problems observed in this group of cows (De Souza et al., 2022).

The management of infection caused by *N. caninum* requires the detection and separation of infected animals, adoption of appropriate breeding practices, vector control, and implementation of sanitary and biosecurity measures (Villa et al., 2022). Moreover, it is crucial to adopt strict sanitary measures at all stages of the embryo transfer process. Techniques such as the polymerase chain reaction are effective tools for identifying the presence of viruses in embryos, uterine fluids, and lavage fluids (Rufino et al., 2006). In addition, the exposure of bovine embryos to infectious agents during *in vitro* production may pose a potential risk of spreading infectious diseases (González Altamiranda et al., 2020). Therefore, it is essential to monitor and prevent possible viral contamination in embryos to ensure the health and integrity of herds (Smirnova et al., 2012).

### Nutritional management

Another important factor is body condition score (BCS). An adequate balance in carbohydrate intake is essential for maintaining pregnancy and preventing pregnancy loss. Metabolic factors such as the excessive formation of ketone bodies and increased blood urea nitrogen may be a threat to pregnancy (Szelényi et al., 2023).

Although, Middleton, Minela, Pursley (2019) and Santos et al. (2023) did not observe any interference in pregnancy loss rates according to BCS, Thangavelu et al. (2015) showed a higher pregnancy loss rate in cows with a low BCS, reaching 9.1%, compared with females with a high BCS, which had a pregnancy loss rate of 1.9%. Furthermore, cows with a low BCS have a longer interval from calving to first estrus and are more frequently subjected to pregnancy loss (Spitzer et al., 1995). Female cattle with a BCS between 2–3 had a higher rate of pregnancy loss, corresponding to 14.91%, whereas cows with a BCS of 3 (1 to 5 points described by Lowman, Scott, Somerville, 1976) had a lower rate of pregnancy loss.

Moreover, the effect of BCS on the rate of late pregnancy loss was most substantial between days 43 and 53 after conception (Jones, Lamb, 2008). Grimard et al. (2006), reported that the incidence of late embryonic mortality was higher in high milk-producing cows and in cows with BCS ≥ 2.5 compared to cows with BCS < 2. Lima et al. (2022) observed that cows that lost their body condition during days 28–56 of gestation had a higher rate (11.6%) of embryonic loss than cows that maintained (4.7%) or gained (5.7%) their body condition during this period. In addition, pregnancy loss rates have been observed to be relatively low, ranging from 3% to 5%, with primiparous females having a lower probability of pregnancy losses (ultrasound performed between 30 and 80 days) than multiparous cows (Zobel et al., 2011; Carvalho et al., 2014).

Energy balance rather than physical condition or reproductive history is an accurate indicator for predicting pregnancy loss in primiparous and multiparous dairy cows, which have similar rates of pregnancy loss with normal and low BCS (Carvalho et al., 2014). Dahl et al. (2020) noted a deterioration in embryonic quality in animals under unfavorable circumstances and in multiparous animals. Furthermore, females that deteriorated their body condition during early lactation showed significant changes in serum lipid levels and increased serum levels of non-esterified fatty acids, suggesting a molecular response in cumulus cells that may have an impact on embryo quality. and fertility (Ruebel et al., 2022).

Maintaining an adequate energy balance is especially important because it directly affects reproductive function, fertility, ovulation, embryo quality, and pregnancy maintenance. Feeding strategies that aim to meet specific nutritional needs at different stages of the reproductive cycle, such as the postpartum period, are essential to ensure reproductive health and minimize pregnancy loss rates (Jones; Lamb, 2008; Middleton, Minela, Pursley (2019).

### Using the CL classification to select recipients

The success of ET depends on factors associated with the embryo and recipient cow, or even the interaction between these factors, the embryo-uterine environment, and the corpus luteum of the recipient. Recipient cows are one of the main factors (along with embryo selection discussed in the previous section) that determine successful pregnancy establishment after ET (Thomson et al., 2021). Initially, in the development of the ET technique, the presence of the CL, which is an evidence of successful ovulation identified by transrectal palpation, was sufficient. The identification of the CL using B-mode ultrasound demonstrated a sensitivity of 86.2% and a specificity of 70.3% (Gómez-Seco et al., 2017). Therefore, many commercial-scale transfer programs recommend only the identification of CL (Pontes et al., 2011; Morotti et al., 2014; Pellegrino et al., 2016; Baruselli et al., 2018; Lovarelli, Bacenetti, Guarino, 2020). However, it is known that evaluating the presence of CL in a recipient's ovary may be sufficient to assess the quality of the CL and consequently support pregnancy.

Although some studies have indicated that the CL size may be important for the maintenance of pregnancy (Gonella-Diaza et al., 2018; Velho et al., 2022), others have not confirmed that the CL size is a mandatory aspect of pregnancy establishment (Pugliesi et al., 2019; Thomson et al., 2021; Santos et al., 2023). Therefore, this issue requires further investigation, although there is a tendency to prioritize animals with CL.

Furthermore, the use of collor Doppler ultrasonography to evaluate blood perfusion in the CL has been intensively investigated. Several studies have demonstrated a direct relationship between the CL blood perfusion and pregnancy maintenance in FTET programs (Pinaffi et al., 2015; Pugliesi et al., 2018; 2019; Santos et al., 2023; Rossignolo et al., 2023). Thus, cows and heifers with greater blood perfusion in the CL have higher serum concentrations of P4, and consequently, higher pregnancy rates (Gómez-Seco et al., 2017; Fontes, Oosthuizen, 2022).

**Figure 1:**
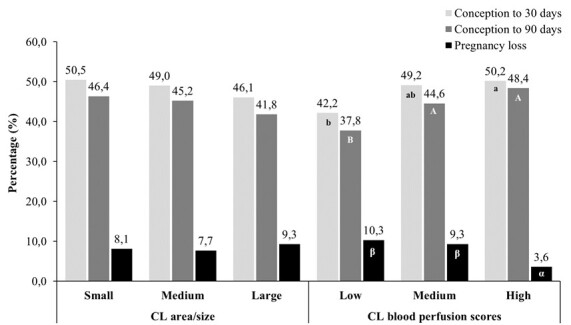
Effect of corpus luteum (CL) area/size (small < 3 cm2; medium > 3 and < 4 cm2; and large > 4 cm^2^) and blood flow score (low < 40% of CL; medium > 45% and < 50% of CL; and high > 55% of CL) on the conception rate in embryo recipients submitted to a timed embryo transfer (TET) program. Different lowercase (a-b), uppercase (A-B) or Greek letters (α-β) for the same variable indicate statistically significant differences (P < 0.05). There was no effect of CL area/size on conception rates at 30 and 90 days or on pregnancy loss.

A recent study evaluated a total of 1,700 Brangus recipients to determine whether area and luteal blood perfusion of corpus luteum (CL) may have any impact on the conception rate and the occurrence of pregnancy loss in a large-scale timed embryo transfer (TET) program (Santos et al., 2023). This study considered commercial IVP embryos in which all recipients with at least one CL received an embryo on the seventh day after ovulation. Each recipient was evaluated by B-mode ultrasound to determine the CL area (cm2) into small (< 3 cm2), medium (> 3 and < 4 cm2), and large (> 4 cm2), in addition to evaluation in color Doppler mode to classify luteal blood perfusion into low (vascularization < 40% of the CL), medium (vascularization > 45% and < 50%), and high score (vascularization > 50%). The results are expressed in Figure 1 and were interesting because it showed that the CL area/size did not affect (P > 0.1) the results of conception or pregnancy loss, but luteal blood flow determined an increase (P < 0.05) in the conception rate and a reduction (P < 0.05) in pregnancy loss. Therefore, strategically, this study showed that the use of Doppler ultrasound to evaluate recipients allows for a more accurate classification of the reproductive efficiency in embryo recipients. Positively impacting to increase the conception rate and reducing the occurrence of pregnancy loss, probably due to the better quality of the CL in the maintenance of pregnancy. All the results mentioned above demonstrate that CL assessment is necessary to predict possible pregnancy success. However, it is still necessary to use different techniques to predict good recipients of bovine embryos on a large scale, and possibly when different aspects are measured/addressed (CL size, CL perfusion, and serum progesterone dosage), the reliability seems to increase.

### Paternal contribution to gestational loss

Among the factors that contribute to gestational loss in cattle, paternal effects have been highlighted as important. This has been highlighted in studies involving dairy and beef livestock production (Franco et al., 2020; Jena et al., 2021; Pohler et al., 2021). However, reproductive success is highly variable and is influenced by maternal and paternal factors. Maternal characteristics, including subspecies, parity, reproductive tract size, reproductive management strategies regarding estrus expression and detection, uterine environment, hormonal secretion, corpus luteum quality, and numerous other factors, can affect conception, pregnancy, and gestational loss (Pohler et al., 2021). However, despite the numerous maternal factors involved in establishing pregnancy, the contribution of bulls to reproductive failure has rarely been investigated and is often completely neglected.

The establishment of pregnancy in cattle is a complex process that encompasses ovulation, fertilization, blastocyst formation, growth into an elongated conceptus, pregnancy recognition signaling, and the development of the embryo and placenta (Ortega et al., 2018). Failure of any of these events can compromise embryonic development and gestational success. Paternal factors that determine gestational success act mainly in early pregnancy events (Starbuck et al., 2004) and have a significant influence on placental and pregnancy losses (Pohler, Oliveira, 2024).

Chromosomal abnormalities caused by spermatogenesis-related disorders may be associated with recurrent embryonic mortality. A study evaluating chromosomal defects in the semen of a group of men with recurrent pregnancy loss, with (fertile group) or without (infertile group) eventual success in pregnancy through assisted reproduction, reported a higher incidence of aneuploidy in the sperm from the infertile group than in the fertile group of men (Cheung et al., 2019). Furthermore, transcriptomic evaluation of fresh bovine semen identified transcripts that were substantially associated with fertilization and embryogenesis, with a greater abundance of THSD4 and PAG5 among the identified PAG proteins, both with strong associations with placental development (Selvaraju et al., 2017). Kropp et al. (2017) analyzed embryos fertilized with either a high- or low-fertility Holstein bull, and RNA-sequencing analysis revealed that 98 genes were differentially expressed. A total of 65 genes were upregulated in high-fertility bull-derived embryos, and 33 genes were upregulated in low-fertility-derived embryos. Furthermore, evaluation of the epigenetic signature of spermatozoa between high- and low-fertility bulls revealed 76 differentially methylated regions.

Murine embryos with only a maternal genetic contribution presented an underdeveloped trophectoderm in the tissues of the conceptus, and embryos with only a paternal genetic contribution had a well-proliferated trophectoderm (Surani et al., 1987). A study conducted with parthenogenetic bovine embryos (embryos without a paternal genome; Pohler et al., 2021) found that the trophectoderm was well-developed up to 30 days of gestation, but no site of embryo attachment to the endometrium was found. Furthermore, the secretion of pregnancy-associated glycoproteins (PAG) and interferon-stimulated genes was not found in the maternal circulation. These results suggest that paternal genetics are necessary for post-elongation attachment of the embryo to the endometrium.

To monitor the occurrence of gestational loss, in addition to ultrasound examination, the concentration of circulating PAG has been widely used, as it allows pregnancy diagnosis before 30 days of the conceptus, covering an earlier period and better reflecting the probability of pregnancy maintenance (Pohler et al., 2013, 2016; Franco et al., 2018). Using PAG quantification and ultrasound at 30 and 60 d, the paternal effect (eight Angus bulls) was evaluated for pregnancy loss in 658 cows subjected to fixed-time insemination (TAI; Franco et al., 2020). Overall, early embryonic death (between 24 and 30 days) was 5.54%, with values ranging from 1.8 to 11.7%, whereas late embryonic death was 6.7%, with values ranging from 2.3 to 12.6%. These results reinforce the importance of bulls’ contribution to the maintenance and establishment of pregnancy in cattle.

Heat stress negatively affects the reproductive performance of cattle and genetic differences in thermotolerance are known to occur. Generally, *Bos indicus* embryos are more thermotolerant than *Bos taurus* embryos (Hernandez-Ceron et al., 2004). This scenario is evident in females and is repeated in bulls. The use of Gir bulls (*Bos indicus*) to inseminate lactating Holstein cows resulted in a higher pregnancy rate and reduced pregnancy loss compared to Holstein bulls (*Bos taurus*; Pegorer et al., 2007). Therefore, in addition to the other factors mentioned in this topic, the father's genetic group also stands out as another factor that can contribute to mitigating pregnancy loss, especially in herds that suffer the most from heat stress. Furthermore, it is believed that there is a link between the large variation in pregnancy rates among bulls when cows express estrus before TAI (Franco et al., 2018, 2020) with the genetic/molecular characteristics of the sperm. Thus, there is a need for a better understanding of the molecular and genetic components of sperm, as well as the interaction between sperm and the female reproductive tract.

Finally, all the aforementioned selection criteria are extremely important for increasing conception rates on farms. Furthermore, several factors, such as seasonality and animal management, can affect the final results. In Chart 1, we summarize the strategies that can reduce EEM.

**Chart 1:** Possible solution strategies for early pregnancy loss considering the donor's embryonic classification, recipient selection, and paternal contribution.

**Solution Strategies for Early Pregnancy Losses**	**Embryo** **Selection**	Always prioritize Grade I quality embryos, regardless of *in vitro* or *in vivo* produced.			**Reference**
					Farin, Slenning, Britt (1999), Erdem et al. (2020), Demetrio et al. (2020)
	**Recipients** **Selection**	**Sanity Management** Vaccination and control with BoHV-1, BVDV, Leptospira *spp*., and Neospora *ssp*	**Nutritional Management** Select recipients with BCS above 2.5 to 4 points (scale 1-5).	**CL Classification** The presence of CL is required. CL perfusion is more important than diameter/dimensions.	Rufino et al. (2006), Smirnova et al. (2012), Aono et al. (2013)González Altamiranda et al. (2020), Jones, Lamb (2008)Thangavelu et al. (2015), Moraes et al. (2018), Pugliesi et al. (2018, 2019), Santos et al. (2023)Rossignolo et al. (2023)
	**Paternal Contribution**	Genetically proven bulls, preferably with real proof of progeny Use of suitable tours for each climate/region Nutritional and Sanity Management			Franco et al. (2018, 2020), Kropp et al. (2017), Pohler et al. (2021)
	**Results**	Better results are expected when all of the above are instituted in a bovine embryo transfer			

## Conclusion

Considering the economic impact of gestational loss on cattle farming, it is necessary to understand the mechanisms that lead to embryonic loss. Current knowledge refers to the consideration of basic aspects, such as nutrition, health, appropriate classification of embryos and recipients, and paternal effects, as the major points to minimize gestational losses. Despite the common situation of trying a single factor responsible for gestational failure, we highlight the importance of all conditions involved in cattle production, including animal welfare, to improve reproductive efficiency and minimize embryonic loss.
